# A Comparison of Impulse Oscillometry and Spirometry by Percent Predicted in Identifying Uncontrolled Asthma

**DOI:** 10.3390/arm93040025

**Published:** 2025-07-18

**Authors:** Chalerm Liwsrisakun, Chaicharn Pothirat, Athavudh Deesomchok, Pilaiporn Duangjit, Warawut Chaiwong

**Affiliations:** Division of Pulmonary, Critical Care and Allergy, Department of Internal Medicine, Faculty of Medicine, Chiang Mai University, Chiang Mai 50200, Thailand; chalerm.liw@cmu.ac.th (C.L.); chaicharn.p@cmu.ac.th (C.P.); athavudh.d@cmu.ac.th (A.D.); pilaiporn.th@cmu.ac.th (P.D.)

**Keywords:** impulse oscillometry, spirometry, asthma control, diagnostic, assessment

## Abstract

**Highlights:**

**What are the main findings?**

- Spirometry does not always correlate closely with asthma control.
- The IOS showed a greater ability to detect asthma control than spirometry.
- R5-R20 ≥ 200 %-predicted is the best point for identifying uncontrolled asthma.

**What are the implications of the main findings?**

- IOS can be used to assess asthma control.

**Abstract:**

Background: The role of impulse oscillometry (IOS) in evaluating asthma control remains a challenge because the interpretation varies by many factors, including ethnicity. We aimed to assess the diagnostic contribution of spirometry and IOS, established from reference equations, in the detection of uncontrolled asthma. Methods: This retrospective study was conducted in adult asthma subjects with normal spirometry. Uncontrolled asthma was defined as an Asthma Control Test (ACT) score ≤ 19. Receiver operating characteristic (ROC) curves were plotted to compare the diagnostic abilities of the %-predicted of heterogeneity of resistance at 5 Hz and 20 Hz (R5-R20) and the %-predicted of forced expiratory volume in the first second (FEV_1_) in detecting uncontrolled asthma. Multivariable risk regressions were performed to identify the %-predicted of R5-R20 as a predictor for uncontrolled asthma. Results: The %-predicted of R5-R20 demonstrated a superior diagnostic ability for detecting uncontrolled asthma compared to the %-predicted FEV_1_, with the area under the ROC curves (AuROC) = 0.939 vs. 0.712, respectively, *p* < 0.001. The %-predicted R5R20 of ≥200 showed the highest AuROC for detecting uncontrolled asthma with an adjusted risk ratio of 10.86 (95%CI; 3.77, 31.29; *p* < 0.001). Conclusions: IOS demonstrated better diagnostic ability for detecting uncontrolled asthma than spirometry.

## 1. Introduction

Asthma is one of the major chronic respiratory diseases that cause recurring episodes of wheezing, breathlessness, chest tightness, and coughing [1]. The worldwide prevalence of asthma ranges from 1% to 18% [1]. In Chiang Mai, Thailand, Pothirat et al. found the prevalence of asthma among adults aged over 40 years was 10.1% [2].

The goals of asthma care are to achieve and maintain asthma control while minimizing the long-term risk of asthma [1]. However, up to 60% of the patients still have poorly controlled asthma, despite being treated with controller medication [3,4,5]. Therefore, the assessment of asthma control is an essential part of asthma care [6]. According to asthma guidelines, the assessment of asthma control is based on physician evaluations, questionnaires, and lung function tests [1]. However, over- or under-rating of asthma control may occur due to physicians’ or patients’ assessments. Consequently, non-invasive and reliable tools for assessing asthma control are still required.

The most common lung function test is spirometry. However, the updated guidelines state that the forced expiratory volume in the first second (FEV_1_), forced vital capacity (FVC), and the average expired flow over the middle-half (25–75%) of the FVC maneuver (FEF 25–75%) from spirometry did not correlate well with asthma symptoms [1]. Impulse oscillometry (IOS) is a new, non-invasive method for measuring lung function [7]. It requires only tidal breathing to measure airway resistance and airway reactance. Previous studies have shown that IOS variables are related to asthma symptoms and can be used to measure asthma control [8,9,10,11,12,13,14,15,16,17,18], particularly the heterogeneity of resistance at 5 Hz and 20 Hz (R5-R20) [8,9,17,18]. However, comparisons between spirometry and IOS for detecting asthma control remain challenging. Takeda et al. demonstrated that IOS correlated better with clinical symptoms and asthma control than spirometry [10]. Moreover, the updated review suggested that peripheral airway impairment diagnosed by the reference equation of R5-R20 was associated with poor asthma control [19]. Nevertheless, some studies indicated that spirometry and IOS measurements were equally useful as potential markers of asthma control [13,20]. Additionally, a previous study found that IOS did not demonstrate sufficient discriminative capacity to classify patients according to the degree of asthma control [21]. As mentioned previously, FEV_1_ from spirometry remains the preferred lung function measure in current global asthma guidelines [1]. These discrepant results might be from the interpretation of the tests because the parameters of spirometry and IOS depended on many factors, including age, sex, height, weight, and ethnicity [22,23]. Thus, more studies are required to compare the percentages of predicted values of spirometry and IOS parameters for detecting asthma control in different countries. Therefore, this study aimed to assess the diagnostic contributions of spirometry and IOS, established from reference equations derived from the Thai population, in distinguishing between uncontrolled and well-controlled adult asthmatic subjects.

## 2. Materials and Methods

### 2.1. Study Design and Population

This retrospective study was conducted at the Lung Health Center, Faculty of Medicine, Chiang Mai University, Chiang Mai, Thailand. The study included adult asthmatic subjects aged over 20 years who fulfilled all the following criteria: 1. non-smoking; 2. normal spirometry, defined as FEV_1_/FVC ≥ lower limit of normal (LLN) (z-score > −1.645) and FVC ≥ LLN (z-score > −1.645); 3. having stable symptoms for more than six weeks; 4. using asthma controller medication regularly for at least three months. Their diagnosis of asthma has been previously made by pulmonologists in accordance with the GINA guidelines [1]. The spirometry results met the American Thoracic Society (ATS)/European Respiratory Society (ERS) standards [22], and IOS results adhered to ERS guidelines [24]. Data were collected from measurements conducted between July 2019 and June 2020. Baseline characteristics, including age, sex, body mass index (BMI), underlying diseases, smoking status, age at disease onset, duration of disease, controller medication used, and Asthma Control Test (ACT) scores, were also recorded. Uncontrolled asthma was defined as an ACT score ≤ 19 [25]. This study received approval from the Research Ethics Committee of the Faculty of Medicine, Chiang Mai University [Institutional Review Board (IRB) approval number: MED-2568-0035, date of approval: 17 January 2025]. Written informed consent was waived due to the retrospective nature of the study.

### 2.2. Lung Function Test

On-treatment IOS parameters, including resistance at 5 Hz (R5), resistance at 20 Hz (R20), heterogeneity of resistance at 5 Hz and 20 Hz (R5-R20), reactance at 5 Hz (X5), area under reactance (AX), and frequency resonance (Fres), were measured using an IOS machine (Master Screen IOS, Viasys GmbH, Hoechberg, Germany). R5-R20 was the marker of small airway disease, which was the index of interest in our study. Thai IOS reference equations [23] were used to calculate the %-predicted values for all IOS parameters. On-treatment spirometry values, including FVC, FEV_1_, FEV_1_/FVC, and FEF 25–75%, were measured using a spirometer (Vmax Encore 22, CareFusion, Hoechberg, Germany). The Global Lung Initiative (GLI) 2012 reference equations for the Southeast Asian subgroup were used to calculate the %-predicted values and z-scores for all spirometry parameters [26].

### 2.3. Study Size Estimation

The sample size calculation was based on data from a previous study [17], which included 46 poorly controlled and 96 controlled asthma patients. The area under the receiver operating characteristic (AuROC) curves of R5-R20 for detecting uncontrolled asthma was 0.91. The AuROC of FEV_1_ for detecting uncontrolled asthma was derived from another study, which reported it as 0.58 [13]. The alpha level and power were set at 0.01 and 80%, respectively. Therefore, we needed to enroll 109 asthma subjects (35 uncontrolled and 74 controlled) in this study.

### 2.4. Statistical Analysis

Continuous data were expressed as means and standard deviations (SD). Non-normally distributed data were presented as medians and interquartile ranges (IQR). Categorical data were shown as counts and percentages. Independent sample *t*-tests and Mann–Whitney U tests were used to compare baseline characteristics, IOS, and spirometry parameters between groups for normal and non-normally distributed data, respectively. Fisher’s exact test was used to compare categorical data between groups. The correlations among ACT scores, IOS parameters, and spirometric values were assessed using Spearman’s correlation coefficient analysis. Weak, moderate, and strong correlations were interpreted according to the criteria: |r| < 0.3 (weak), 0.3 < |r| < 0.7 (moderate), and |r| > 0.7 (strong) [27]. ROC curves were plotted to compare the diagnostic abilities of the %-predicted values of R5-R20 and %-predicted values of FEV_1_ to detect uncontrolled asthma by calculating the AuROC with a 95% confidence interval (CI). Contingency tables were created to compute the sensitivity, specificity, positive likelihood ratio (+LR), negative likelihood ratio (−LR), diagnostic odds ratio, AuROC, and Youden index from various values of the %-predicted of R5-R20 to identify the optimal cut-off point for detecting uncontrolled asthma. Univariable risk regressions were conducted to identify %-predicted values of R5-R20 as a predictor of uncontrolled asthma. Additionally, multivariable risk regressions were performed to ascertain %-predicted values of R5-R20 as a predictor of uncontrolled asthma while adjusting for confounding factors, including age, gender, BMI, allergic rhinitis (AR), diabetes mellitus (DM), history of asthma exacerbations (AE) in the previous year, and the inhaled corticosteroid (ICS) dose. Results were expressed as a risk ratio (RR) and adjusted RR with a 95% CI for RR for univariable and multivariable risk regressions, respectively. A *p*-value < 0.05 was considered statistically significant. All statistical analyses were performed using STATA version 16.

## 3. Results

One hundred and nine subjects, with 35 uncontrolled and 74 well-controlled asthmatics, were included in this study. No significant differences were observed between the two groups, including age, sex, age of disease onset, disease duration, family history of asthma, daily ICS dose, oral medication use, and comorbidities. BMI was significantly higher in the uncontrolled group compared to the well-controlled group. The proportion of patients with a history of AE in the previous year was significantly higher in the uncontrolled group. ACT scores were significantly lower in the uncontrolled group. Additional data are presented in Table 1.

Spirometric values, including absolute and %-predicted values, and z-scores for FVC, FEV_1_, and FEF 25–75%, were significantly lower in the uncontrolled group than in the well-controlled group. In contrast, the absolute value and z-score of the FEV_1_/FVC ratio were not different between the two groups. Additional data are presented in Table 2.

Significantly increased absolute values of IOS parameters, including R5, R20, R5-R20, Fres, and AX, were observed in the uncontrolled group. A significant decrease in the absolute value of X5 was observed in the uncontrolled group compared to the well-controlled group. Additionally, significantly increased %-predicted values of all IOS parameters, except for R20, were recorded in the uncontrolled group. Additional data are presented in Table 3.

Correlations between ACT scores, IOS parameters, and spirometric parameters are shown in Table 4. Low-to-moderate correlations were observed between IOS parameters and ACT scores, with the %-predicted value of R5-R20 demonstrating the strongest correlation (r = −0.643). Low-to-moderate correlations were also observed between spirometric parameters and ACT scores, with the %-predicted FEV_1_ demonstrating the strongest correlation (r = 0.302). Further details are provided in Table 4.

**Figure 1:**
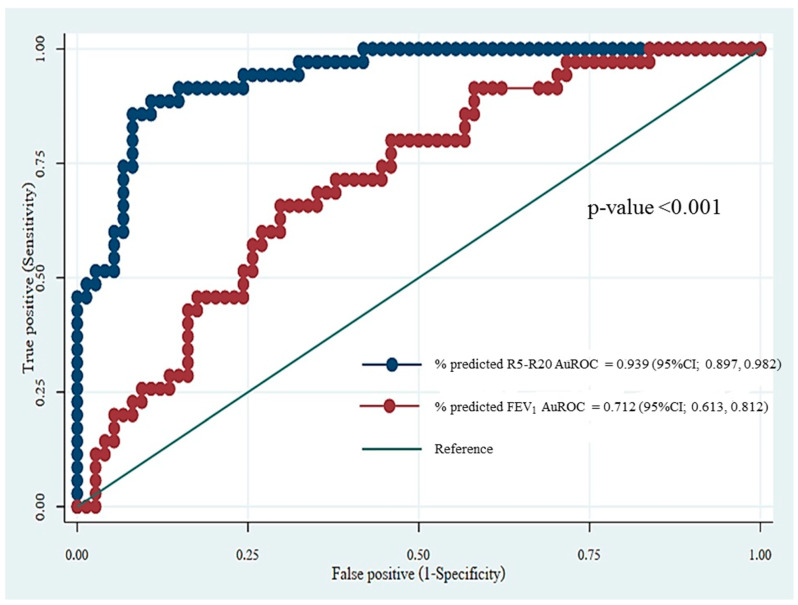
Comparison of receiver operating curve between %-predicted value of R5-R20 and %-predicted value of FEV_1_ for detection of uncontrolled asthma. Abbreviations: R5-R20, heterogeneity of resistance between R5 and R20; FEV_1_, forced expiratory volume in the first second.

The %-predicted value of R5-R20 demonstrated superior diagnostic ability for detecting uncontrolled asthma compared to the %-predicted value of FEV_1_ (AuROC = 0.939 vs. 0.712, respectively, *p* < 0.001) (Figure 1).

The sensitivity, specificity, +LR, −LR, diagnostic odds ratio, and AuROC for various cut-off points of %-predicted R5-R20 for identifying uncontrolled asthma are presented in Table 5. The optimal cut-off point was ≥200, which demonstrated the highest AuROC of 0.88 and a Youden index of 76.4, with a sensitivity of 88.6% and a specificity of 87.8% for detecting uncontrolled asthma. Additional data are presented in Table 5.

In an explanatory analysis focusing on the role of %-predicted R5-R20 in detecting uncontrolled asthma after adjusting for potential confounding factors, the multivariable analysis, after adjusting for age, sex, BMI, AR, DM, history of AE in the previous year, and ICS dose, showed that a %-predicted R5-R20 ≥ 200 was the strongest predictor of uncontrolled asthma, with an adjusted relative risk (RR) of 10.86 (95%CI; 3.77, 31.29, *p* < 0.001). Additional data are presented in Table 6.

## 4. Discussion

This study investigated the advantages of IOS over spirometry in detecting uncontrolled asthma. We found that the %-predicted value of R5-R20 demonstrated superior diagnostic ability for detecting uncontrolled asthma compared to the %-predicted value of FEV_1_. After adjusting for potential confounding factors in a multivariable analysis, we determined that a %-predicted R5-R20 ≥ 200 was the strongest predictor of uncontrolled asthma.

We analyzed the correlation between the %-predicted value of R5-R20, %-predicted value of FEV_1_, and asthma control, as measured by the ACT. We observed that the correlations of the %-predicted value of R5-R20 and %-predicted value of FEV_1_ with ACT scores were only a low-to-moderate degree. The strongest correlation was observed between the %-predicted R5-R20 and ACT score (r = −0.643), while the correlation between the %-predicted FEV_1_ and ACT score was only 0.302. Our results were consistent with previous studies demonstrating low-to-moderate correlations between lung function and asthma control, with correlation coefficients ranging from 0.27 to 0.58 [3,10,28]. Prior research had also reported a weak correlation between asthma symptoms and objective measures of airway obstruction, such as spirometry [29].

We observed an increase in IOS parameters, particularly the %-predicted value of R5-R20, in uncontrolled asthma, which was consistent with previous studies indicating significantly higher R5-R20 values in poorly controlled asthma compared to well-controlled asthma [16,17,30]. Furthermore, in subjects with normal spirometry, the %-predicted values of all IOS parameters except R20 were significantly higher in the uncontrolled group compared to the well-controlled group. This finding supported previous research demonstrating that the pathological changes in patients with uncontrolled asthma primarily affected small airways rather than large airways [18]. Additionally, the superior diagnostic ability of the %-predicted values of R5-R20 for detecting uncontrolled asthma compared to the %-predicted values of FEV_1_ aligned with previous findings [19]. In our study, the AuROC for the %-predicted R5-R20 and %-predicted FEV_1_ in detecting uncontrolled asthma were 0.939 and 0.712, respectively, which were comparable to previous studies reporting AuROC values for R5-R20 ranging from 0.810 to 0.911 and for FEV_1_ ranging from 0.580 to 0.718 for detecting poor asthma control [8,9,13,17,18]. Although spirometry remains the gold standard for asthma monitoring [18], its limitations, particularly the requirement for maximum forced expiratory maneuvers, have been noted. Moreover, FEV_1_ primarily reflects a large airway function rather than a small airway function [31].

In an explanatory research model, we also investigated the role of the %-predicted value of R5-R20 in detecting uncontrolled asthma after adjusting for potential confounding factors, including age, sex, BMI, AR, DM, history of AE in the previous year, and ICS dose. The strongest predictor of uncontrolled asthma was a %-predicted R5-R20 of ≥ 200. Previous studies had shown that small airway dysfunction (SAD), as measured by IOS, was more prevalent than when measured by spirometry [32,33]. The significance of SAD detection in asthma was its association with poor symptom control [17,18,19] and more frequent asthma exacerbations [30,34]. Furthermore, Cottini et al. suggested that SAD, measured using oscillometry, could serve as a potentially treatable trait in asthma management [34]. Therefore, IOS parameters, particularly the %-predicted value of R5-R20, can be useful for assessing the level of asthma control. Additionally, IOS can be used instead of spirometry to detect asthma control, especially in asthma subjects with normal spirometry.

A strength of our study is that it not only identifies the cut-off values for the %-predicted R5-R20 in detecting uncontrolled asthma but also shows that this level of the parameter is the best predictor of uncontrolled asthma despite normal spirometry. This confirms the benefits of IOS as a useful tool for detecting uncontrolled asthma and, because of its ease of performance, should be added to routine assessment of asthma control in clinical practice. However, this study has some limitations. First, because airway resistance and reactance can be affected by abnormal spirometry, including airway obstruction and lung restriction, we only included subjects with normal spirometry. Therefore, the cut-off values for the %-predicted R5-R20 in detecting uncontrolled asthma in subjects with abnormal spirometry may differ. Second, we used Thai prediction equations for IOS parameters. Thus, our cut-off points for detecting uncontrolled asthma in other settings should be interpreted cautiously due to potential differences in reference values. Moreover, the diagnostic performance of the %-predicted R5-R20 cut-off (≥200) is derived from a single-center retrospective dataset without external validation. Therefore, a prospective or multi-center validation cohort would strengthen the generalizability of the findings. Third, although multivariable risk regression models were included, the impact of possible factors such as medication adherence, inhalation technique, or the socioeconomic status of asthma control was not accounted for in the analysis, even though these factors could independently influence ACT scores and lung function. Fourth, although ACT is used as the gold standard for defining “uncontrolled asthma” in this study, there are limitations and potential subjectivity elements associated with ACT, particularly its dependence on patient recall and perception. Thus, results should be interpreted with caution due to the limitations of measuring asthma control using ACT.

## 5. Conclusions

In adult asthma patients with normal spirometry, the %-predicted value of R5-R20 demonstrated superior diagnostic accuracy for detecting uncontrolled asthma compared to the %-predicted FEV_1_. A %-predicted R5-R20 ≥ 200 is the optimal cut-off value for identifying uncontrolled asthma. Therefore, IOS can be a useful tool for assessing asthma control.

## Figures and Tables

**Table 1:** Baseline characteristics of asthma subjects (n = 109).

Clinical Characteristics	Uncontrolled (n = 35)	Well-Controlled (n = 74)	*p*-Value
Age (years)	56.7 ± 15.9	53.6 ± 15.4	0.333
Female sex n (%)	27 (77.1)	57 (70.3)	0.500
Body mass index (BMI)	28.6 ± 3.9	24.9 ± 3.9	<0.001
Age of disease onset (year) (median, IQR)	34.0 (27.0, 60.0)	36.0 (22.0, 54.0)	0.393
Duration of disease (year) (median, IQR)	11.0 (3.0, 33.0)	12.0 (3.0, 27.0)	0.997
Family history of asthma (yes)	18 (51.4)	42 (56.8)	0.682
ACT score	16.8 ± 2.7	23.0 ± 1.6	<0.001
Inhaled medication used			0.001
ICS	5 (14.3)	3 (4.1)	
ICS + LABA	20 (57.1)	65 (87.8)	
ICS + LABA + LAMA	10 (20.6)	6 (8.1)	
Daily dose of ICS			0.116
Low	13 (37.1)	43 (58.1)	
Medium	19 (54.3)	25 (33.8)	
High	3 (8.6)	6 (8.1)	
Oral medication used			
Antileukotriene	23 (65.7)	45 (60.8)	0.676
Oral bronchodilator	3 (8.6)	2 (2.7)	0.325
Omalizumab	2 (5.7)	2 (2.7)	0.592
Comorbidities n (%)			
Rhinitis	30 (85.7)	65 (87.8)	0.765
Hypertension	11 (31.4)	19 (25.7)	0.647
Diabetes mellitus	6 (17.1)	5 (6.8)	0.169
History of AE in the previous year	14 (40.0)	8 (10.8)	0.001

Note: Data are mean ± standard deviation (SD) unless otherwise stated.

**Table 2:** Absolute value, %-predicted, and Z-score of spirometric parameters.

Spirometry Parameters	Uncontrolled (n = 35)	Well-Controlled (n = 74)	*p*-Value
FVC (L)	2.29 ± 0.64	2.74 ± 0.68	0.002
%-predicted of FVC	92.0 ± 13.3	99.9 ± 13.7	0.006
z-score of FVC	−0.59 ± 0.87	−0.01 ± 0.97	0.002
FEV_1_ (L)	1.77 ± 0.54	2.16 ± 0.56	0.001
%-predicted of FEV_1_	84.9 ± 11.2	94.6 ± 14.4	<0.001
z-score of FEV_1_	−1.01 ± 0.71	−0.31 ± 1.00	<0.001
FEV_1_/FVC (%)	78.0 ± 5.1	79.1 ± 6.5	0.382
z-score of FEV_1_/FVC	−0.94 ± 0.69	−0.65 ± 0.88	0.097
FEF 25–75% (L/sec)	1.59 ± 0.78	2.07 ± 0.89	0.008
%-predicted of FEF 25–75%	69.3 ± 17.6	84.8 ± 27.8	0.003
z-score of FEF 25–75%	−1.10 ± 0.64	−0.58 ± 0.98	0.005

Note: Data are mean ± standard deviation (SD). Abbreviations: FVC, forced vital capacity; FEV_1_, forced expiratory volume in the first second; FEF 25–75%, forced expiratory flow at 25–75% of FVC; L, liter.

**Table 3:** Absolute value and %-predicted of IOS parameters.

IOS Parameters	Uncontrolled (n = 35)	Well-Controlled (n = 74)	*p*-Value
**Absolute value**			
R5 (cmH_2_O/L/s)	5.99 ± 1.64	3.79 ± 1.08	<0.001
R20 (cmH_2_O/L/s)	3.88 ± 0.94	3.17 ± 0.95	<0.001
R5-R20 (cmH_2_O/L/s)	1.92 (1.55, 2.42)	0.53 (0.30, 0.84)	<0.001
X5 (cmH_2_O/L/s)	−2.11 (−3.02, −1.52)	−1.09 (−1.50, −0.77)	<0.001
Fres (Hz)	22.24 ± 4.42	14.61 ± 3.33	<0.001
AX (cmH_2_O/L)	17.43 (11.97, 26.66)	3.78 (2.21, 6.58)	<0.001
**%-Predicted value**			
R5	120.4 ± 30.5	91.9 ± 25.4	<0.001
R20	94.6 ± 22.3	92.4 ± 28.6	0.687
R5-R20	325.6 (236.8, 432.6)	96.9 (50.4, 162.9)	<0.001
X5	170.6 (140.9, 237.6)	108.2 (75.6, 147.1)	<0.001
Fres	151.3 ± 29.2	112.8 ± 28.4	<0.001
AX	376.6 (233.3, 576.0)	107.9 (56.9, 158.3)	<0.001

Note: Data are mean ± standard deviation (SD) or median (interquartile range, IQR). Abbreviations: R5, resistance at 5 Hz; R20, resistance at 20 Hz; R5-R20, heterogeneity of resistance between R5 and R20; Fres, resonant frequency; X5, reactance at 5 Hz; AX, the area under reactance curve between 5 Hz and resonant frequency.

**Table 4:** Correlation between spirometric parameters, IOS parameters, and Asthma Control Test (ACT) Score.

		ACT	%-Predicted Value								
			FVC	FEV_1_	FEF _25–75%_	R5	R20	R5-R20	X5	Fres	AX
	ACT	1.000									
**%-Predicted value**	FVC	0.260 *	1.000								
	FEV_1_	0.302 *	0.897 *	1.000							
	FEF _25–75%_	0.259 *	0.379 *	0.705 *	1.000						
	R5	−0.323 *	−0.205 *	−0.234 *	−0.149	1.000					
	R20	−0.008	−0.076	−0.084	−0.013	0.811 *	1.000				
	R5-R20	−0.643 *	−0.201 *	−0.247 *	−0.221 *	0.514 *	0.016	1.000			
	X5	−0.333 *	−0.268 *	−0.236 *	−0.109 *	0.664 *	0.304 *	0.499 *	1.000		
	Fres	−0.447 *	−0.314 *	−0.301 *	−0.206 *	0.502 *	0.098	0.724 *	0.415 *	1.000	
	AX	−0.537 *	−0.267 *	−0.302 *	−0.236 *	0.703 *	0.247	0.795 *	0.776 *	0.711 *	1.000

Note: *, *p*-value < 0.05. Abbreviations: FVC, forced vital capacity; FEV_1_, forced expiratory volume in the first second; FEF 25–75%, forced expiratory flow at 25–75% of FVC; R5, resistance at 5 Hz; R20, resistance at 20 Hz; R5-R20, heterogeneity of resistance between R5 and R20; Fres, resonant frequency; X5, reactance at 5 Hz; AX, the area under reactance curve between 5 Hz and resonant frequency.

**Table 5:** Diagnostic performances of %-predicted levels of R5-R20 in the detection of uncontrolled asthma.

Cut-Off	Sensitivity (95%CI)	Specificity (95%CI)	+LR	−LR	AUC	Youden Index
≥150	94.3 (80.8, 99.3)	70.3 (58.5, 80.3)	3.17 (2.21, 4.54)	0.08 (0.02, 0.32)	0.82 (0.76, 0.89)	64.6
≥200	88.6 (73.3, 96.8)	87.8 (78.2, 94.3)	7.28 (3.90, 13.60)	0.13 (0.05, 0.33)	0.88 (0.82, 0.95)	76.4
≥250	74.3 (56.7, 87.5)	93.2 (84.9, 97.8)	11.0 (4.61, 26.20)	0.28 (0.16, 0.49)	0.84 (0.76, 0.92)	67.5

Note: AUC from cut-off based ROC. Abbreviations: R5-R20, heterogeneity of resistance between R5 and R20; FEV1, forced expiratory volume in the first second; +LR, positive likelihood ratio; −LR, negative likelihood ratio; AUC, area under the curve.

**Table 6:** Univariable and multivariable analysis for identifying R5-R20 as a predictor of uncontrolled asthma.

Factors	Univariable Analysis		Multivariable Analysis	
	RR (95%CI)	*p*-Value	Adjusted RR (95%CI)	*p*-Value
R5-R20 ≥ 200% of predicted value	13.37 (5.09, 35.11)	**<0.001**	10.86 (3.77, 31.29)	**<0.001**
Age	1.01 (0.99, 1.03)	0.329	0.99 (0.97, 1.02)	0.547
Female gender	1.28 (0.66, 2.49)	0.466	1.56 (0.61, 4.00)	0.355
Body mass index (BMI)	1.09 (1.05, 1.14)	**<0.001**	1.14 (1.04, 1.25)	**0.005**
Allergic rhinitis	0.88 (0.41, 1.89)	0.752	1.14 (0.38, 3.36)	0.818
Diabetes mellitus	1.84 (0.99, 3.43)	0.053	1.64 (0.65, 4.14)	0.291
History of AE in the previous year	2.64 (1.62, 4.29)	**<0.001**	1.98 (0.91, 4.32)	0.087
ICS dose				
Low	Ref.		Ref.	
Medium	1.86 (1.03, 3.34)	**0.037**	0.98 (0.46, 2.09)	0.965
High	1.44 (0.51, 4.06)	0.495	0.91 (0.23, 3.67)	0.894

Note: Ref. stands for reference group. Abbreviations: R5-R20, heterogeneity of resistance between R5 and R20; AE, acute exacerbation; ICS, inhaled corticosteroid.

## Data Availability

The data that support the findings of this study are available from the corresponding author upon reasonable request.
